# Time-locked auditory cortical responses in the high-gamma band: A window into primary auditory cortex

**DOI:** 10.3389/fnins.2022.1075369

**Published:** 2022-12-08

**Authors:** Jonathan Z. Simon, Vrishab Commuri, Joshua P. Kulasingham

**Affiliations:** ^1^Department of Electrical and Computer Engineering, University of Maryland, College Park, College Park, MD, United States; ^2^Department of Biology, University of Maryland, College Park, College Park, MD, United States; ^3^Institute for Systems Research, University of Maryland, College Park, College Park, MD, United States; ^4^Department of Electrical Engineering, Linköping University, Linköping, Sweden

**Keywords:** phase locked response, medial geniculate body, high frequency, envelope following response, cortical FFR

## Abstract

Primary auditory cortex is a critical stage in the human auditory pathway, a gateway between subcortical and higher-level cortical areas. Receiving the output of all subcortical processing, it sends its output on to higher-level cortex. Non-invasive physiological recordings of primary auditory cortex using electroencephalography (EEG) and magnetoencephalography (MEG), however, may not have sufficient specificity to separate responses generated in primary auditory cortex from those generated in underlying subcortical areas or neighboring cortical areas. This limitation is important for investigations of effects of top-down processing (e.g., selective-attention-based) on primary auditory cortex: higher-level areas are known to be strongly influenced by top-down processes, but subcortical areas are often assumed to perform strictly bottom-up processing. Fortunately, recent advances have made it easier to isolate the neural activity of primary auditory cortex from other areas. In this perspective, we focus on time-locked responses to stimulus features in the high gamma band (70–150 Hz) and with early cortical latency (∼40 ms), intermediate between subcortical and higher-level areas. We review recent findings from physiological studies employing either repeated simple sounds or continuous speech, obtaining either a frequency following response (FFR) or temporal response function (TRF). The potential roles of top-down processing are underscored, and comparisons with invasive intracranial EEG (iEEG) and animal model recordings are made. We argue that MEG studies employing continuous speech stimuli may offer particular benefits, in that only a few minutes of speech generates robust high gamma responses from bilateral primary auditory cortex, and without measurable interference from subcortical or higher-level areas.

## Introduction

Primary auditory cortex plays a key role in the human brain’s processing of sounds, being a major gateway between auditory subcortical areas, including the inferior colliculus (midbrain) and thalamus, and higher order auditory cortical areas, including secondary auditory areas, associative auditory areas, and language areas. While the neurophysiology of primary auditory cortex has been studied for decades in animal models, there are still many unanswered questions. One of the hallmarks of primary auditory cortex in animal models is its sluggishness compared to subcortical areas, since its typical neurons time-lock^1^ to acoustic modulations only up to a few tens of Hz (Lu et al., 2001; Joris et al., 2004), though at the same time it does respond very reliably (temporally) to brief acoustic features, with a spiking precision of milliseconds both for punctate features (Phillips and Hall, 1990; Heil and Irvine, 1997) and ongoing spectrotemporally dynamic features (Elhilali et al., 2004).

Less is known about temporal processing in *human* primary auditory cortex, where neurophysiological recording techniques for healthy subjects are restricted to non-invasive methods, primarily electroencephalography (EEG) and magnetoencephalography (MEG). Neither EEG nor MEG has very fine spatial resolution (typically a few centimeters) and so may not be able to distinguish different neural sources based purely on their anatomical origin. Both, however, have sufficient temporal resolution to distinguish typical response latencies of primary auditory cortex (∼40 ms) from subcortical (shorter latency) and non-primary (longer latency) auditory areas.

Beyond these commonalities, EEG and MEG have distinctive strengths and weaknesses. EEG is sensitive to neural sources throughout the brain at both low frequencies (tens of Hz) and high frequencies (hundreds of Hz) (Kraus et al., 2017; White-Schwoch et al., 2019). It is therefore relatively straightforward to record time-locked activity from any auditory area of the brain, but it may be difficult to distinguish contributions from multiple areas, at least without additional information (e.g., response latency, which can be used to distinguish between the sources giving rise to the auditory P1 and N1 components). In contrast, MEG is insensitive to subcortical neural sources (Hämäläinen et al., 1993), though not entirely unresponsive, as seen below. Perhaps counterintuitively, this insensitivity gives MEG an advantage over EEG, by allowing recordings from auditory cortical sources without substantial subcortical interference (Ross et al., 2020). Nevertheless, MEG responses from different auditory cortical areas can still interfere with each other.

Another consideration is that EEG’s sensitivity to most auditory sources holds for both low and high frequencies, but because of MEG’s cortical bias and because cortical responses are usually sluggish, MEG typically only captures cortical sources at low frequencies. An important counterexample, however, is the case of fast (∼100 Hz) auditory time-locked cortical responses (Hertrich et al., 2012; Coffey et al., 2016). At these frequencies there are few, if any, cortical sources aside from primary auditory cortex. In this sense, MEG recordings of fast time-locked auditory cortical responses act as an exquisite window into primary auditory cortex, without interference from subcortical or other cortical areas. Therefore, it may be especially suited for questions regarding how primary auditory cortical responses are affected by cognitive processes, whether modulated by top-down neural activity (e.g., selective attention or task-specific processing) or supplemented by super-auditory aspects of the stimulus (e.g., processing of speech sounds using language-based information).

One newly established method to analyze neural responses to continuous speech (Hamilton and Huth, 2018) is temporal response function (TRF) analysis (Lalor et al., 2009; Ding and Simon, 2012). TRFs are an effective tool to disambiguate neural sources based on their characteristic latencies, as will be discussed below.

## Results

Fast (∼100 Hz) cortical time-locked auditory responses are typically investigated using one of two different stimulus paradigms. The more time-honored paradigm is the frequency following response (FFR) (Kraus et al., 2017), for which a typical stimulus is either acoustically simple, such as click trains or amplitude modulated tones (e.g., Gorina-Careta et al., 2021), or consists of many repetitions of a short but more complex stimulus, such as a single syllable (e.g., Coffey et al., 2016).

**FIGURE 1:**
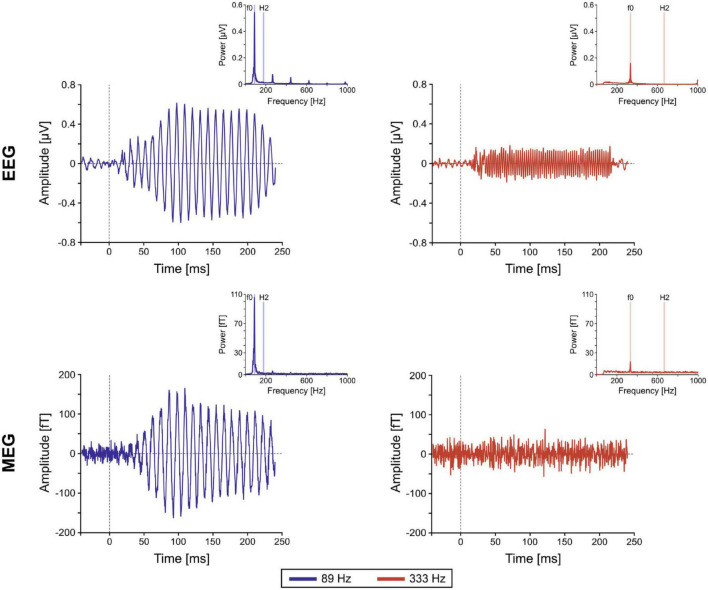
Example frequency following responses (FFRs). Grand-averaged FFR time course and spectral representations (insets) of single-channel EEG and magnetoencephalography (MEG) elicited in the high gamma frequency range (89 Hz; blue) and the very high gamma range (333 Hz; red). It can be shown that the very high gamma frequency (333 Hz; red) FFR is almost entirely subcortical for both EEG and MEG. In contrast, the high gamma frequency (89 Hz; blue) FFR is almost entirely cortical for MEG and a mix of cortical and subcortical for EEG [from Gorina-Careta et al. (2021), Figure 1].

The well-established FFR paradigm (or really, family of paradigms, including the envelope following response; EFR) has been used to great effect with EEG to investigate midbrain responses to acoustic stimuli. Near 100 Hz, midbrain sources dominate the EEG FFR over cortical sources, and well above 100 Hz there is little to no cortical EEG FFR contribution at all (Coffey et al., 2019). Until the MEG FFR investigations of Coffey et al. (2016), however, it was not widely appreciated how substantial the cortical FFR contributions might be near 100 Hz. In this seminal paper, the investigators presented the 120-ms syllable/da/, synthesized with a 98 Hz fundamental frequency in the vowel portion, for 14,000 repetitions (sufficient to also obtain responses from subcortical sources despite the cortical bias of MEG). The cortical responses, whose sources were consistent with primary auditory cortex, were prominent and showed a significant lateralization to the right hemisphere, with a longer latency profile compared to subcortical components. This work firmly established the measurability of distinct cortical contributions to the FFR near 100 Hz. In comparison, Gorina-Careta et al. (2021) demonstrated that the MEG FFR at the much higher frequency of 333 Hz (15,200 tone-burst repetitions) originated solely from subcortical sources (Figure 1). Note that both these studies demonstrate that, while MEG is not incapable of measuring high frequency FFR from subcortical sources, the number of repetitions required is considerable, with an associated experimental design cost (e.g., limited to a small number of stimulus types).

One of the limitations of the FFR paradigm is that accessing the different latencies of distinct sources may not be straightforward, since the FFR is ultimately just the evoked response to a sustained stimulus: a linear sum of overlapping responses from multiple sources with different latencies (Teichert et al., 2022). A more recently developed paradigm uses neural responses to continuous speech, such as individual sentences (e.g., Hertrich et al., 2012) or longer narrated story passages (e.g., Kulasingham et al., 2020). The use of the continuous speech stimulus paradigm, combined with TRF analysis, sidesteps this temporal overlap issue by deconvolving the sustained response from the stimulus, which often allows direct comparison of neural source peak latencies. Though typical uses of TRF analysis employ the slow (<10 Hz) acoustic envelope as the stimulus feature with which to deconvolve (Di Liberto et al., 2015; Cervantes Constantino and Simon, 2018), the TRF methodology generalizes well to other stimulus features (Brodbeck and Simon, 2020). This includes responses from high frequency stimulus features processed in subcortical areas (Maddox and Lee, 2018; Polonenko and Maddox, 2021).

**FIGURE 2:**
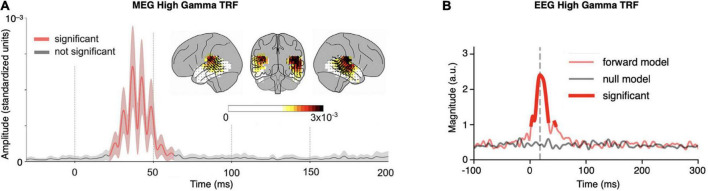
Example high gamma temporal response functions (TRFs). **(A)** High frequency (70–200 Hz) magnetoencephalography (MEG) TRF from 6 mins of continuous speech. The grand-averaged amplitude of TRF source localized current-dipole vectors, averaged across voxels in the cortical ROI, is shown (±standard error across subjects; red indicates amplitude significantly greater than noise). The TRF has a peak latency of ∼40 ms and oscillates with a frequency of ∼80 Hz (note that since only the TRF amplitude is shown, and not signed current values, signal troughs and peaks both appear as peaks). Inset: the distribution of TRF current-dipole vectors in the brain at each voxel at the moment of the maximum response; color represents response amplitude (standardized units) and arrows represent TRF current-dipole orientations [modified from Kulasingham et al. (2020), Figure 3]. **(B)** High frequency (70–200 Hz) EEG TRF from 40 mins of continuous speech. The grand-averaged magnitude of the Hilbert transform of the TRF, averaged across channels, is shown; bright red indicates magnitude significantly greater than the null model. The TRF magnitude significantly exceeds that of the null model in two latency ranges: between 2 and 33 ms with a peak at 18 ms (dominantly subcortical; grey dashed line), and between 44 and 46 ms with a peak at 45 ms (dominantly cortical) [modified from Kegler et al. (2022), Figure 3].

High frequency (70–200 Hz) MEG TRFs were first investigated by Kulasingham et al. (2020) using only 6 mins of continuous speech as the stimulus. Responses source-localized to bilateral primary auditory cortex, with a small but significant lateralization to the right hemisphere (Figure 2A). The peak latency of the cortical response, 40 ms, is consistent with a primary auditory cortical origin. Analysis additionally revealed that frequencies contributing to time-locking fell off substantially above 100 Hz. This demonstration that such a short recording can reveal responses localized to primary auditory cortex serves several purposes. It allows future experiments to include multiple stimulus conditions (e.g., presenting stimuli under different task conditions or at different SNRs), and at the same time ensures that the responses do not contain measurable subcortical interference.

High frequency (70–200 Hz) EEG TRFs with cortical contributions have also been recently investigated by Kegler et al. (2022). These TRFs show a pair of peaks with distinguishable latencies allowing inference of separate sources, each with a separate anatomical origin and auditory processing role (analogous to traditional P1 and N1 peaks arising from separate cortical sources). In this case, the earlier peak at 18 ms is consistent with a subcortical origin, and the later peak at 45 ms is consistent with a dominantly cortical origin (Figure 2B).

It should not be surprising that invasive iEEG recordings had already demonstrated similar high gamma time-locked cortical responses almost a decade earlier (Brugge et al., 2009; Steinschneider et al., 2013), using click trains and isolated speech sounds. What is surprising is that such responses could be seen even non-invasively. The most robust time-locked high gamma iEEG responses are seen in primary auditory cortex, specifically posteromedial Heschl’s gyrus (Nourski, 2017), but smaller time-locked high gamma responses are also seen in other auditory cortical areas. As such, iEEG remains a premiere electrophysiological method for obtaining responses known to originate in primary auditory cortex, but only for a fraction of subjects relative to those eligible for MEG or EEG recordings.

## Discussion

As indicated above, a physiological window into human primary auditory cortex allows the investigation of the extent to which primary auditory cortex is influenced by higher order cortical areas. How, and under which circumstances, are primary auditory cortical responses modulated by top-down neural activity, or affected by language-specific non-auditory features of the stimulus? A related question is to what extent subcortical auditory areas might be influenced by cortical processing. Neither can be answered without first identifying the specific sources of neural activity (e.g., midbrain vs. thalamus vs. primary auditory cortex) being modulated by distant cortical activity.

Using MEG, Hartmann and Weisz (2019) demonstrated that the FFR near 100 Hz from right hemisphere primary auditory cortex is modulated by intermodal (auditory vs. visual) attention. Most FFR investigations use EEG, which is well-suited to separate responses from primary auditory cortex from those originating in other cortical areas, but, as indicated above, has difficulty in separating auditory subcortical and primary auditory cortical contributions. Intriguing results include: modulation of the EEG FFR by selective attention for frequencies near 100 Hz but not above 200 Hz (Holmes et al., 2018); modulation by overall level of attention near 150 Hz (Price and Bidelman, 2021); and, at 100 Hz, modulation by whether a continuous-speech masker is in a known vs. unknown (but acoustically similar) language (Presacco et al., 2016; Zan et al., 2019). There has also been a report of selective attentional modulation of subcortical auditory responses to continuous speech (Forte et al., 2017); the result has not yet been replicated, however, and due to the specialty of the analysis method it is as yet difficult to rule out entirely whether the result might be due to cortical response leakage.

More recently, using EEG with a continuous speech stimulus, Kegler et al. (2022) demonstrated that the high gamma EEG TRF arising from a combination of subcortical and primary auditory cortical sources (illustrated in Figure 2B) is modulated by word-boundary effects. This is strong evidence that a linguistic (super-acoustic) feature can modulate either primary auditory cortical or auditory subcortical processing (or both). Kulasingham et al. (2022) have also recently demonstrated that the high gamma MEG TRF, originating solely from bilateral primary auditory cortex, is indeed modulated by selective attention, using re-analysis of previously published data (Kulasingham et al., 2021).

There is additional evidence that human primary auditory cortical responses exhibit modulation arising from other cortical areas, but the effects are subtle. Using iEEG and employing selective attention to one of two competing talkers, O’Sullivan et al. (2019) did not observe modulation of cortical responses in Heschl’s gyrus (the anatomical location of primary auditory cortex), while, in contrast, they did find modulation in non-primary areas, as expected. Using a similar paradigm to investigate the role of selective attention on MEG low frequency cortical TRFs, Brodbeck et al. (2018), did see evidence of significant TRF modulation at short latencies consistent with a primary auditory cortex origin (in addition to the expected strong modulation at longer latencies), but only under limited conditions.

In animal studies, top-down (task-dependent) modulation of neural activity in primary auditory cortex has been seen as far back as two decades ago (Fritz et al., 2003). Despite the robustness and reproducibility of these results, however, the effect size is nevertheless small, and it has not been clear until recently whether such modulations would ever be observable non-invasively.

What is the physiological origin of the high gamma time-locked responses from primary auditory cortex? Two theories have been put forward. The first concerns the physics underlying the generators of EEG and MEG signals, which are dominantly driven by dendritic currents produced by synaptic inputs (Hämäläinen et al., 1993; Buzsaki et al., 2012), i.e., the same mechanisms that also give rise to the local field potential (LFP). For primary auditory cortex, the most significant neural input is the spiking output of the medial geniculate body (MGB) of the thalamus, whose spiking rates can reach up to 100 Hz (Miller et al., 2002), and whose thalamocortical fibers show ensemble-wide time-locking up to 300 Hz (Steinschneider et al., 1998), in animal models. A second theory, strongly tied to the first, is that the spikes of primary auditory cortex, which can only fire at rates well below 100 Hz, can nevertheless fire with temporal precision of the order of milliseconds (Elhilali et al., 2004). It has been recently shown by Downer et al. (2021) that these precise but infrequent spikes are actually highly synchronous across the local population, even to the point of acting as a time-locked *population* model for fast acoustic features (almost up to 200 Hz). Indeed, Gnanateja et al. (2021) recently demonstrated a connection between both these explanations, using intracortical FFR (90–140 Hz) recordings from multiple species, to show both an LFP FFR and a multi-unit (spiking) FFR, in the thalamorecipient layers of primary auditory cortex.

In conclusion, recent advances in auditory neuroscience have opened up new non-invasive windows into the neurophysiology of primary auditory cortex. Using EEG FFR techniques, responses are dominantly subcortical but also contain strong contributions from primary auditory cortex at frequencies near 100 Hz. Using MEG FFR techniques, responses are dominantly from primary auditory cortex for frequencies near 100 Hz (though at higher frequencies subcortical responses can also be detected given sufficient recording time). EEG TRF studies have the potential to show both auditory subcortical and primary auditory cortical contributions to the time-locked high gamma responses to continuous speech, but, unlike FFR, segregated in time/latency. Finally, MEG time-locked high gamma TRF studies may hold great promise in isolating primary auditory cortical responses from other areas, due to its insensitivity to subcortical sources and its ability to differentiate competing cortical sources in both time and anatomical location.

## Data availability statement

The original contributions presented in this study are included in the article/supplementary material, further inquiries can be directed to the corresponding author.

## Author contributions

JZS wrote the initial draft of the manuscript. All authors contributed to the interpretations of results and discussions, were involved in manuscript revision, and approved the final version.

## Footnotes

1 We employ the term “time-locked” neural responses rather than “phase-locked” since phase is only defined when the coupled stimulus/response is analyzed in a narrow frequency band. The term “time-locking”, sometimes called “neural tracking” when applied to low frequency responses to speech, applies equally well to narrowband and broadband cases.
